# Differential Expression of Claudin Family Members during Osteoblast and Osteoclast Differentiation: Cldn-1 Is a Novel Positive Regulator of Osteoblastogenesis

**DOI:** 10.1371/journal.pone.0114357

**Published:** 2014-12-05

**Authors:** Fatima Z. Alshbool, Subburaman Mohan

**Affiliations:** 1 Musculoskeletal Disease Center, Jerry L Pettis VA Med Ctr, Loma Linda, CA 92357, United States of America; 2 Department of Medicine, Loma Linda University, Loma Linda, CA 92354, United States of America; 3 Department of Biochemistry, Loma Linda University, Loma Linda, CA 92354, United States of America; 4 Department of Physiology, Loma Linda University, Loma Linda, CA 92354, United States of America; 5 Department of Pharmacology, Loma Linda University, Loma Linda, CA 92354, United States of America; Emory University School of Medicine, United States of America

## Abstract

Claudins (Cldns), a family of 27 transmembrane proteins, represent major components of tight junctions. Aside from functioning as tight junctions, Cldns have emerging roles as regulators of cell proliferation and differentiation. While Cldns are known to be expressed and have important functions in various tissues, their expression and function in bone cells is ill-defined. In this study, the expression of Cldns was examined during osteoblast and osteoclast differentiation. The expression of Cldn-1, -7, -11, and -15 was downregulated during early stages of osteoclast differentiation, whereas Cldn-6 was upregulated. Moreover, the expression of several Cldns increased 3–7 fold in fully differentiated osteoclasts. As for osteoblasts, the expression of several Cldns was found to increase more than 10-fold during differentiation, with some peaking at early, and others at late stages. By contrast, only expression of Cldn-12, and -15 decreased during osteoblast differentiation. In subsequent studies, we focused on the role of Cldn-1 in osteoblasts as its expression was increased by more than 10 fold during osteoblast differentiation and was found to be regulated by multiple osteoregulatory agents including IGF-1 and Wnt3a. We evaluated the consequence of lentiviral shRNA-mediated knockdown of Cldn-1 on osteoblast proliferation and differentiation using MC3T3-E1 mouse osteoblasts. Cldn-1 knockdown caused a significant reduction in MC3T3-E1 cell proliferation and ALP activity. Accordingly, expression levels of cyclinD1 and ALP mRNA levels were reduced in Cldn-1 shRNA knockdown cells. We next determined if Cldn-1 regulates the expression of Runx-2 and osterix, master transcription factors of osteoblast differentiation, and found that their levels were reduced significantly as a consequence of Cldn-1 knockdown. Moreover, knocking down Cldn-1 reduced β-catenin level. In conclusion, the expression of Cldn family members during bone cell differentiation is complex and involves cell type and differentiation stage-dependent regulation. In addition, Cldn-1 is a positive regulator of osteoblast proliferation and differentiation.

## Introduction

Osteoporosis is a major public health problem in the U.S. that is characterized by low bone mass and structural deterioration of bone tissue, resulting in increased bone fragility [1], [2]. It can result from failure to produce optimal bone mass during active growth periods and/or the imbalance between bone formation and bone resorption during bone remodeling processes [2]. It is well known that bone formation is mediated by osteoblasts, which are derived from mesenchymal stem cells [3]. On the other hand, bone resorption is mediated by osteoclasts, which are generated from hematopoietic stem cells derived from a macrophage/monocyte lineage [4], [5]. In terms of osteoblast and osteoclast differentiation, such processes are divided into multiple stages and regulated by temporal and sequential expression of several genes [6]–[8]. Although a number of systemic hormones and local regulatory factors have been shown to regulate bone formation and resorption, our understanding of the molecular pathways that modulate the formation and function of osteoblasts and osteoclasts is, to date, still limited. Thus, the identification of novel genes that participate in regulating osteoblast and osteoclast differentiation and functions is imperative for advancing our understanding of the pathogenesis of osteoporosis.

Tight junctions, which are composed of several types of transmembrane proteins including Claudins (Cldns), junctional adhesion molecule, occludin, and tricellulin [9], are important in the development and maintenance of various tissues [10]. It is noteworthy that there is significant evidence suggesting that Cldns are the principal proteins responsible for the formation of tight junction strands [9]–[11]. The Cldn family of proteins is comprised of 27 members in mouse and human cells with a molecular weight ranging from 20–34 kDa [10], [12], and are divided into two major groups, i.e., “classic” and “non-classic” according to sequence analysis and functional properties of the mouse variants [13]. Moreover, Cldns exhibit complex patterns of expression that is tissue/cell type and developmental stage specific [10], [14], where certain tissues/cells express several Cldns, and others express only one or two [10], [15]. Interestingly, Cldns expression varies within the same tissue in a developmental/differentiation stage dependent fashion [16]. In terms of their regulation, it has been demonstrated that the expression and function of Cldns is controlled by several transcription factors, hormones, and cytokines [14]. As for their function, Cldns act canonically as barriers/pores to regulate paracellular permeability of ions and small molecules, and serve as a fence that divides apical and basolateral domains of plasma membranes [14]. Recently, a distinct role for Cldns has emerged, in which they were shown to serve as mediators of cell signaling. Thus, a “non-canonical function” for Cldns was observed, as they were found to control cell proliferation and differentiation [14], [17]. Even though progress has been made, our knowledge of the expression patterns and function(s) of many Cldn family members in various tissues, their expression, regulation, and role in bone remains ill defined.

It has been shown that bone cells (osteoblasts and osteocytes) form tight junctional structures [18]–[20], and that rat osteoblasts express several Cldns at the mRNA level [21]. Furthermore, recent work by our laboratory have documented that osteoclasts express Cldn-18 and that its deletion, in mice, results in an osteopenia phenotype, i.e., markedly decreased total body bone mineral density, cortical thickness, and trabecular volume [22]. Interestingly, the negative effect of a lack of Cldn-18 on the skeleton was found to be independent of tight junction functions and mediated by increased bone resorption and osteoclast differentiation [22], [23]. While our studies have laid down the foundation for the non-canonical function of Cldn-18 in regulating bone homeostasis, whether other Cldn family members are expressed and have a function during osteoblastogenesis and osteoclastogenesis remains to be investigated. We, therefore, undertook studies to evaluate the expression of Cldn family members during osteoblast and osteoclast differentiation at the mRNA level, and to examine the regulation and function of selected Cldns. Our findings demonstrate that both osteoblasts and osteoclasts express several Cldns and that their expression levels vary depending on cell type and differentiation stage. We have also determined Cldn-1 to be a positive regulator of osteoblast proliferation and differentiation, and is regulated by several osteoregulatory factors. In addition, knocking down Cldn-1 reduced β-catenin level, suggesting that it may be involved in Cldn-1 regulation of osteoblastogenesis.

## Materials and Methods

### Reagents

Ascorbic acid (AA) and β-glycerophosphate (βGP) were purchased from Sigma Chemicals (St. Louis, MO). Minimum essential medium α (α-MEM) and AA free α-MEM were from Life Technologies (Carlsbad, CA). Calf serum (CS) was purchased from Hyclone (Logan, UT). Fetal bovine serum (FBS) was purchased from Atlanta Biologicals (Norcross, GA). Wnt3a, recombinant macrophage colony stimulating factor (M-CSF), and receptor activator of nuclear factor kappa B ligand (RANKL) were from R&D systems (Minneapolis, MN, USA). IGF-1 was a gift from Upjohn pharmacia (Stockholm, Sweden). Cldn-1 specific polyclonal antibody was from cell signaling technology (Danvers, MA). Antibodies to β-actin and β-catenin, and the MISSION shRNA lentiviral particles against Cldn-1 and control non-target short-hairpin RNA (shRNA) lentiviral particles were purchased from Sigma-Aldrich (St. Louis, MO).

### Cell culture

Primary osteoclast precursor or bone marrow macrophages (BMMs) were isolated from femurs and tibias of C57BL/6J mice, as described previously [24], and maintained in α-MEM supplemented with 10% FBS, P/S, and MCSF (25 ng/ml). Twenty four hours later, non-adherent cells (BMMs) were collected and treated with M-CSF (25 ng/ml) and RANKL (50 ng/ml) for different time periods (0, 1, and 6 days); with fresh differentiation media added every 3 days. Primary calvarial osteoblasts were isolated from the calvarias of 3 days old C57BL/6J mice, as previously described [25]. To induce differentiation, calvarial osteoblasts were incubated with AA free α-MEM supplemented with 10% CS, penicillin (P, 100 units/ml), streptomycin (S, 100 µg/ml), βGP (10 mM), and AA (100 µg/ml) for different time periods (0, 4, 6, 8, 13, 19, and 24 days); with fresh differentiation media added every 3 days. All mice were housed in VA Loma Linda Health Care System VMU (Loma Linda, CA, USA) under standard approved laboratory conditions. All animal experiments were performed in compliance with and approved by the Institutional Animal Care and Use Committee of VA Loma Linda Health Care System VMU (Loma Linda, CA, USA). All animals were euthanized using CO2, except 3 days old were euthanized by decapitation. MC3T3-E1 mouse preosteoblast cells were grown in AA free α-MEM supplemented with 10% CS, and P/S. Twenty four hours prior to treatment, cells were incubated in a serum free medium containing AA free α-MEM, 0.1% bovine serum albumin (BSA), and P/S. Cells were then treated with different osteoregulatory agents (BMP-7, IGF-1, vitamin-D3, and Wnt3a) for 72 hrs. All the treatments were made in AA free α-MEM, 0.1% (BSA), and P/S. Both control short-hairpin RNA (shRNA) and Cldn-1 shRNA MC3T3-E1 cells were grown in AA free α-MEM supplemented with 10% CS, and P/S. Prior to treatment, cells were incubated in a serum free medium containing AA free α-MEM, 0.1% bovine serum albumin (BSA), and P/S for 24 hrs. Subsequently cells were treated with βGP (10 mM) and AA (100 µg/ml) for 24 hrs and 6 days.

### RNA extraction and gene expression analysis

RNA was extracted from primary cultures and MC3T3-E1 cells using Trizol and chloroform, and isolation was completed using E.Z.N.A. RNA Isolation Kits (Omega Bio –Tek, Norcross, GA). The purity of RNA was determined by the ratio of the absorbance at 260 and 280 nm. Reverse transcription was accomplished using SuperScript II Reverse Transcriptase (Invitrogen, Carlsbad, CA) and the cDNA were used for real-time RT-PCR. The housekeeping gene peptidylpropyl isomerase A (PPIA) was used as an internal control in the PCR reaction and the fold change compared to control was calculated according to the formula 2^−ΔΔCt^. Primers used for real-time RT-PCR are listed in Table. 1.

**Table 1:** Primer sequences used in real-time RT-PCR.

Gene	Forward (5′-3′)	Reverse (5′-3′)	Ref
**PPIA**	CCATGGCAAATGCTGGACCA	TCCTGGACCCAAAACGCTCC	[45]
**ALP**	ATGGTAACGGGCCTGGCTACA	AGTTCTGCTCATGGACGCCGT	[46]
**Cyclin-D1**	AATGTACTCTGCTTTGCTGAA	ATGAGACCACTAGAGGTCG	[22]
**Osteocalcin**	CTCTCTCTGCTCACTCTGCT	TTTGTAGGCGGTCTTCAAGC	[22]
**Osterix**	AGAGGTTCACTCGCTCTGACGA	TTGCTCAAGTGGTCGCTTCTG	[45]
**Runx-2**	AAAGCCAGAGTGGACCCTTCCA	ATAGCGTGCTGCCATTCGAGGT	[45]
**TRAP**	CACTCAGCTGTCCTGGCTCAA	CTGCAGGTTGTGGTCATGTCC	[22]
**Cldn-1**	GATGTGGATGGCTGTCATTG	CGTGGTGGGTAAGAGGT	[47]
**Cldn-2**	ATGTCCTCGCTGGCTTGTATTAT	GCCATGAAGATTCCAAGCAACTG	[48]
**Cldn-3**	CAGTGTACCAACTGCGTACAAGAC	ACCGGTACTAAGGTGAGCAGAG	[48]
**Cldn-4**	TCGTGGGTGCTCTGGGGAT	GCGGATGACGTTGTGAGCG	[48]
**Cldn-5**	GCTGGCGCTGGTGGCACTCTTT	GCGAACCAGCAGAGCGGCAC	[48]
**Cldn-6**	TGCCCACTCTATCATCCAGGACTTC	AGGCCTGAGGCTGCCCAG	[49]
**Cldn-7**	TTTCATTGTGGCAGGTCTTG	CCAGAAGGACCAGAGCAGAC	[50]
**Cldn-8**	GGCAACCTACGCTCTTCAAA	CAGGGAGTCGTAGACCTTGC	[50]
**Cldn-9**	AAGAGAGAACTGGGGGCTTC	AACGGGAAGGGATGGAGTAG	[50]
**Cldn-10**	ATCTGCGTTACCGATTCCAC	GATCTGAGCCTCCGACTTTG	[51]
**Cldn-11**	CTGCCGAAAAATGGACGAACTG	TGCACGTAGCCTGGAAGGATGA	[49]
**Cldn-12**	ACTGCTCTCCTGCTGTTCGT	TGTCGATTTCAATGGCAGAG	[50]
**Cldn-13**	TAGTGTTGGCCTTCTGATGC	AGCCAAGCAATGGGTTAAAG	[52]
**Cldn-14**	GCTCCTAGGCTTCCTGCTTA	CTGGTAGATGCCTGTGCTGT	[52]
**Cldn-15**	CATCTTTGAGAACCTGTGGTACAGC	GATGGCGGTGATCATGAGAGC	[53]
**Cldn-16**	ATTCATCACCCTGCTCCTTG	AGAGGAGCGTTCGACGTAAA	[50]
**Cldn-17**	TCGTTCTGATTCCAGTGTCC	TCCTCCAAGTTCTCGCTTCT	[52]
**Cldn-19**	CCCAGCACTCCTGTCAATG	GTGCAGCAGAGAAAGGAACC	[54]
**Cldn-20**	GGTACACCAAGGAGATCATAGCG	TACAGGGCTCCTCCAGGTTCATA	[53]
**Cldn-22**	CTTCCGAACGGCAACGCA	CCTCCCGACTTCCTCCTGG	[55]
**Cldn-23**	ACAGGGACACCAGCAAGCTCAA	CGGAGTCACAGGGCAGCGAA	[52]

### Protein extraction and western blotting

Primary calvarial osteoblasts were isolated from 3 days old mice and incubated with AA free α-MEM +10% CS+P/S+ βGP ± AA (100 µg/ml) for 6 days. Both control shRNA and Cldn-1 shRNA MC3T3-E1 cells were grown in AA free α-MEM supplemented with 10% CS, and P/S. Prior to treatment, cells were incubated in a serum free medium containing AA free α-MEM, 0.1% bovine serum albumin (BSA), and P/S for 24 hrs. Subsequently, cells were treated with βGP (10 mM) and AA (100 µg/ml) for 48 hrs. Whole cell extracts were prepared as previously described [22]. Equivalent amounts of protein were resolved on 10% SDS polyacrylamide gel under denaturing conditions and were transferred to a PVDF membrane (Millipore, Billerica, MA). Membranes were blocked with 10% non-fat dry milk dissolved in Tris buffered saline with 0.1% Tween-20 overnight with rotation at 4°C. The following day, membranes were probed with Cldn-1, β-catenin, and β-actin antibodies for 1 hr at room temperature. The membranes were washed and probed with appropriate horseradish peroxidase conjugated secondary antibody (1∶15,000; Sigma-Aldrich; St. Louis, MO). Chemiluminescent substrate (Thermo Fisher Scientific, Waltham, MA) was used to detect the bands.

### Lentivirus transduction

MC3T3-E1 cells were transduced with control shRNA or Cldn-1 shRNA lentiviral particles using a multiplicity of infection (MOI) of 10 in the presence of hexadimethrine bromide (8 µg/ml), as previously described [26]. The following day, the lentiviral particles containing media was changed with fresh media, followed by puromycin selection (10 µg/ml) for 7 days. The knockdown efficiency was evaluated by real-time RT-PCR and western blot.

### Proliferation assay

cells were plated at 3,000 cells/well in 96-well plates with α-MEM (AA free) +10% CS +P/S. The following day, the medium was changed to serum free α-MEM (AA free) +0.1% BSA for 24 hrs, before the cells were treated with βGP ± AA for 48 hrs, rinsed with PBS and frozen at −80°C. Cell proliferation was assessed using a CYQUANT© cell proliferation kit as per the manufacturer's instructions (Life Technologies, Carlsbad, CA). Fluorescence measurements were made using a microplate reader with excitation at 485 nm and emission detection at 530 nm.

### Alkaline phosphatase (ALP) activity assay

cells were plated at 5,000 cells/well in 96-well plates with α-MEM (AA free) +10% CS +P/S. The following day, the medium was changed to serum free α-MEM (AA free) +0.1% BSA for 24 hrs. The cells were then treated with βGP ± AA for 72 hrs, before being rinsed with PBS and permeabilized with ALP activity buffer containing 0.1% triton X-100 in 250 mM NaHCO3. Protein concentration was determined by the BCA method (Thermo Fisher Scientific; Waltham, MA) and ALP activity was detected as previously described [27].

### ALP staining

Cells were incubated with AA free α-MEM +10% CS+P/S+ βGP ± AA (100 µg/ml) for 6 days. Cells were washed with PBS, fixed with 10% formalin for 20 minutes at room temperature, and incubated at 37°C for 30 min in staining buffer containing 50 mM Tris-HCL, pH 8.6, 100 mM NaCl, 5 mM KCl, 1 mM MgCl_2_, 0.8 mg/ml naphthol AS-TR phosphate, and 0.6 mg/ml fast red violet LB diazonium (Sigma Aldrich, St. Louis, MO). Quantification of ALP-stained areas was measured with ImageJ software.

### Statistical Analysis

Data are expressed as the mean ± SEM (n = 4–8) and were analyzed using one-way ANOVA (GraphPad Prism6) for the expression of Cldns during osteoblast and osteoclast differentiation and the Student's t test (Microsoft Excel) for all other experiments. Newman- Keuls was used as a post hoc test.

## Results

### Expression profiles of Cldn family members during osteoclast differentiation

**Figure 1:**
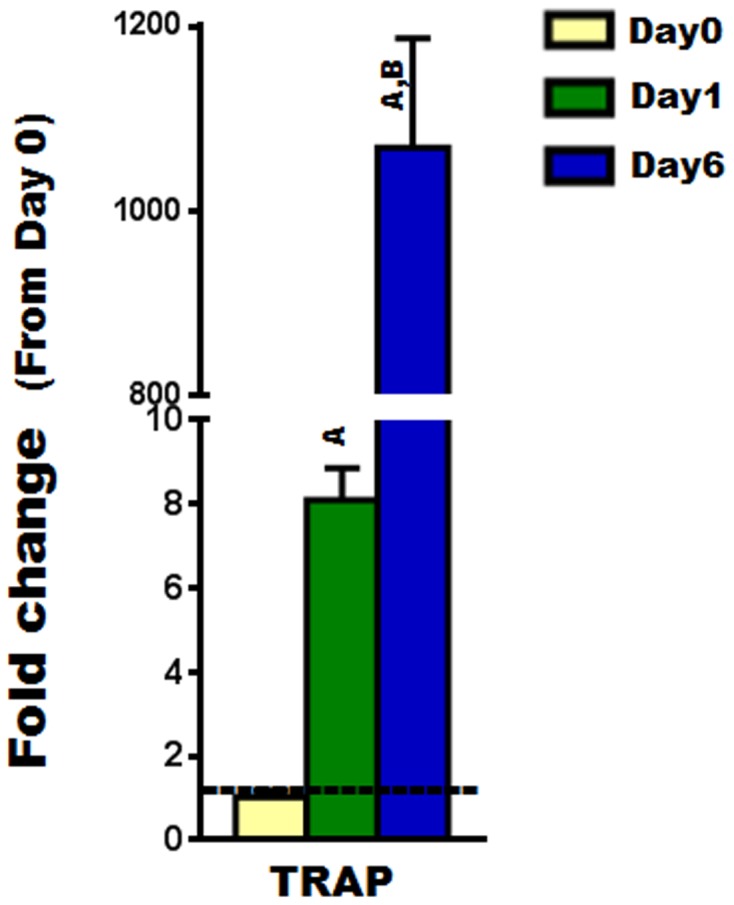
The expression level of tartrate resistant acid phosphatase (TRAP), an osteoclastogenic marker gene, during RANKL induced osteoclast differentiation. The expression level of TRAP during primary osteoclast differentiation was evaluated by real time RT-PCR, at different time points (0, 1, and 6 days). Values are (means ± SEM; n = 4) presented as fold change from day 0 (untreated cells); data were analyzed using one-way ANOVA (GraphPad Prism6). A = <0.05 (from day 0), and B = <0.05 (from day 1).

**Figure 2:**
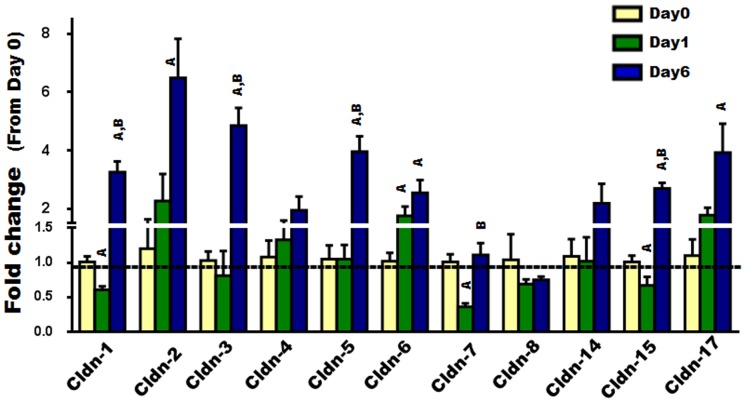
The expression level of “classic” Cldns during RANKL induced osteoclast differentiation. The expression level of “classic” Cldns (Cldn 1-8, -14, -15, and -17) during primary osteoclast differentiation was evaluated by real time RT-PCR, at different time points (0, 1, and 6 days). Values (means ± SEM; n = 4) are presented as fold change from day 0 (untreated cells)), data were analyzed using one-way ANOVA (GraphPad Prism6). A = <0.05 (from day 0), and B = <0.05 (from day 1).

**Figure 3:**
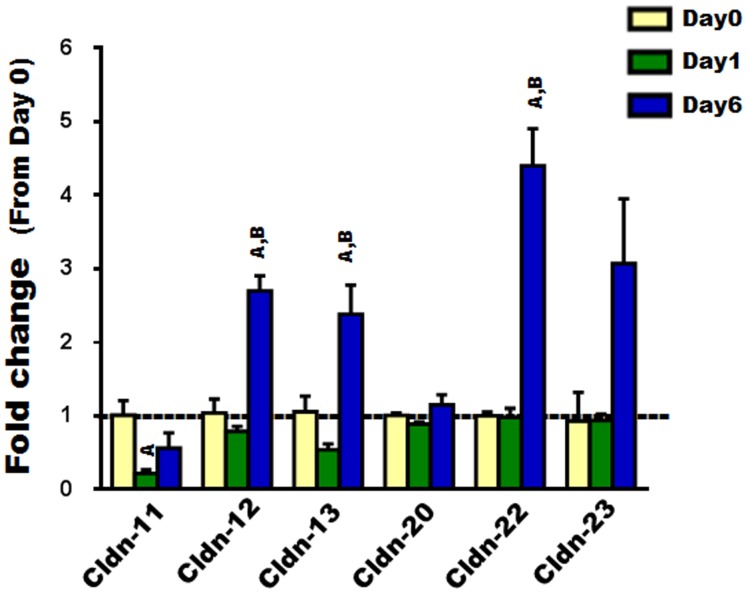
The expression level of “non-classic” Cldns during RANKL induced osteoclast differentiation. The expression level of “non-classic” Cldns (Cldn 11-13, -20, -22, and -23) during primary OC differentiation was evaluated by real time RT-PCR, at different time points (0, 1, and 6 days). Values (means ± SEM; n = 4) are presented as fold change from day 0 (untreated cells), data were analyzed using one-way ANOVA (GraphPad Prism6). A = <0.05 (from day 0), and B = <0.05 (from day 1).

To examine the expression of Cldns during osteoclast differentiation, we used primary osteoclast precursors isolated from bone marrow (i.e., BMMs) and treated them with MCSF and RANKL for different time periods (0, 1, and 6 days). Firstly, osteoclast differentiation was verified by measuring the bone resorption marker gene, TRAP, which is known to increase as osteoclasts differentiate (Fig. 1) [4]. Our findings indicated that some of the “Classic” Cldns including Cldns-1, -7, and -15 were downregulated during early stages (day 1) of osteoclast differentiation, whereas Cldn-6 was upregulated. As for the expression of “Classic” Cldns, several of them (i.e., -1, -2, -3, -5, -6, -15, and -17), were found to be increased 3–7 fold in fully differentiated osteoclasts compared to undifferentiated osteoclasts (day 6 vs. day 0; Fig. 2). As for the remaining “Classic” Cldns (-4,-8, and -14), no significant change in their expression levels was observed (Fig. 2). Of the “non-classic” Cldns, Cldn-11 was downregulated during the early stage (day 1) of osteoclast differentiation; Cldn-12, -13, and -22 were upregulated during late stages (day 6); while no changes in Cldn-20 and -23 mRNA levels were detected (Fig. 3). Taken together, these findings suggest that Cldns are differentially expressed and highly regulated during osteoclast differentiation.

### Expression profiles of Cldn family members during osteoblast differentiation

**Figure 4:**
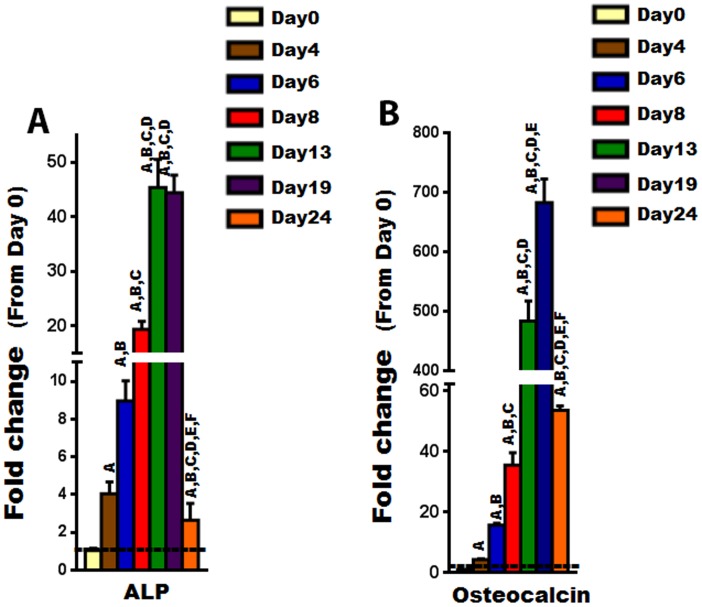
The expression level of osteoblastogenic marker genes during ascorbic acid induced osteoblast differentiation. A) The expression of alkaline phosphatase (ALP), an early stage maker gene of osteoblast differentiation, as determined by real time RT-PCR. B) The expression of osteoclacin, a late stage maker gene of osteoblast differentiation, as determined by real time RT-PCR. Values (means ± SEM; n = 6) are presented as fold change from day 0 (untreated cells), data were analyzed using one-way ANOVA (GraphPad Prism6). A = <0.05 (from day 0), B = <0.05 (from day 4), C = <0.05 (from day 6), D = <0.05 (from day 8), E = <0.05 (from day 13), and F = <0.05 (from day 19).

**Figure 5:**
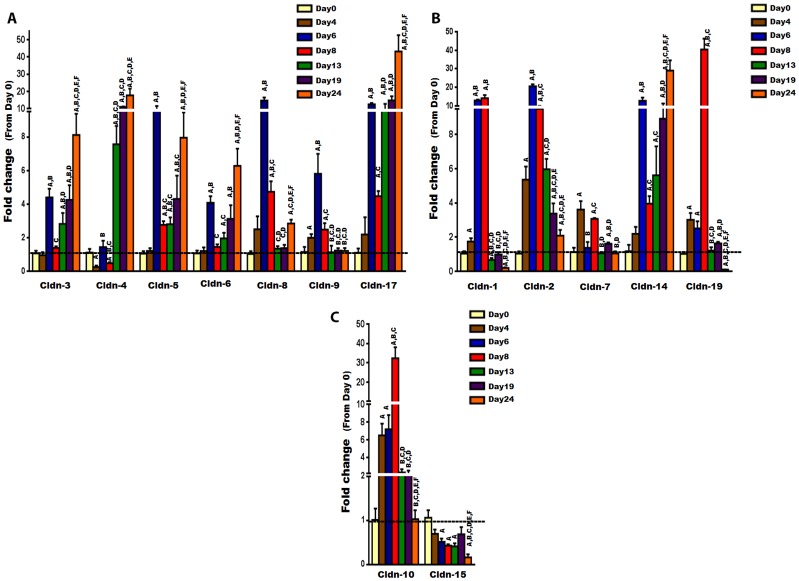
The expression level of “classic” Cldns during ascorbic acid induced osteoblast differentiation. The expression level of “classic” Cldns during primary osteoblast differentiation was measured by real time RT-PCR, at different time points (0, 4, 6, 8, 13, 19, 24 days). A) The expression level of cluster A of the “classic” Cldns, which includes Cldn-3, -4, -5, -6, -8, -9, and -17. B) The expression level of cluster B of the “classic” Cldns, which includes Cldn-1, -2, -7, -14, and -19. C) The expression level of cluster C of the “classic” Cldns, which includes Cldn-10 and -15. Values (means ± SEM; n = 6) are presented as fold change from day 0 (untreated cells), data were analyzed using one-way ANOVA (GraphPad Prism6). A = <0.05 (from day 0), B = <0.05 (from day 4), C = <0.05 (from day 6), D = <0.05 (from day 8), E = <0.05 (from day 13), and F = <0.05 (from day 19).

To investigate the expression of Cldns during osteoblast differentiation, primary osteoblasts were isolated from calvarias of 3 days old mice and differentiated with AA for different time periods (0, 4, 6, 8, 13, 19, and 24 days). The expression of Cldns was then evaluated by real-time RT-PCR. We first confirmed osteoblast differentiation by measuring osteoblast marker genes such as ALP and osteocalcin, which are known to increase, in a time-dependent manner, as osteoblasts differentiate (Fig. 4) [28]. Given that Cldns are divided into “classic” and “non-classic” groups [13], for the sake of simplicity and ease of comprehension, the “classic” ones were subdivided into three clusters based on their phylogenetic tree close proximity, as follows: (1) Cluster A, which includes Cldn-3, -4, -5, -6, -8, -9, and -17; (2) Cluster B, which includes Cldn-1, -2, -7, -14, and -19; and (3) Cluster C, which includes Cldn-10 and -15. On the other hand, the less similar/related Cldns were classified as “non-classic”, and include Cldn-11, -12, -13, -16, -18, -20, -22, and -23. Our results revealed that the expression levels of all “Classic” Cldns were upregulated during osteoblast differentiation, except Cldn-15 which was found to be downregulated (Fig. 5A, B, C). Interestingly, expression levels of Cldn-1, -2, -5, -7 to -10, and -19 were increased significantly during early stages of osteoblast differentiation, i.e., peaked between day 6 and 8, whereas the mRNA levels of Cldn-3, -4, -6, -14, and -17 peaked at late stages (Fig. 5A, B, C). It is noteworthy that several “classic” Cldns were found to be upregulated by more than 10 fold, at certain stages of osteoblast differentiation, including Cldn-1, -2, -4, -5, -8, -10, -14, -17, and -19 (Fig. 5A, B, C).

**Figure 6:**
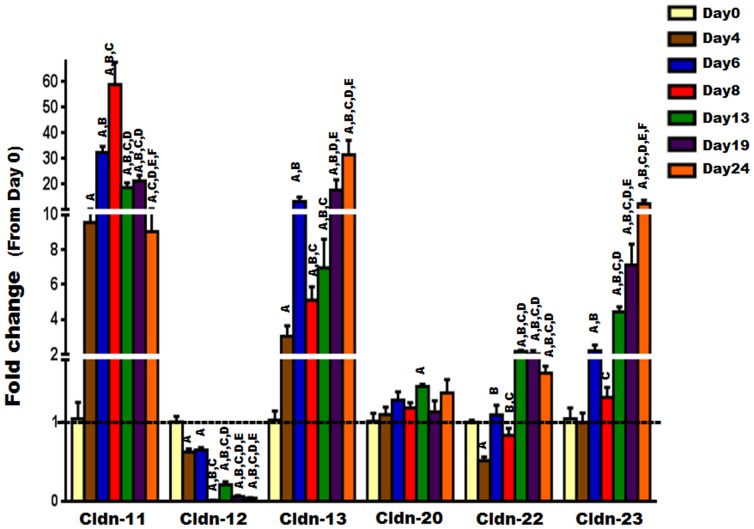
The expression level of “non-classic” Cldns during ascorbic acid induced osteoblast differentiation. The expression level of “non-classic” Cldns during primary osteoblast differentiation was evaluated by real time RT-PCR at different time points (0, 4, 6, 8, 13, 19, 24 days). Values (means ± SEM; n = 6) are presented as fold change from day 0 (untreated cells), data were analyzed using one-way ANOVA (GraphPad Prism6). A = <0.05 (from day 0), B = <0.05 (from day 4), C = <0.05 (from day 6), D = <0.05 (from day 8), E = <0.05 (from day 13), and F = <0.05 (from day 19).

Regarding the “non-classic Cldns”, our results indicate that they exhibit a diverse expression profile. While the expression levels of Cldn-11, -13, -22, and -23 were increased significantly during osteoblast differentiation and peaked during either early or late stages (Fig. 6), those of Cldn-12 decreased significantly (Fig. 6). Moreover, no change in the expression level of Cldn-20 was observed during osteoblast differentiation (Fig. 6). Of note, the mRNA of Cldn-16 was found to be undetectable by real-time RT-PCR, at least under our experimental conditions. Collectively, these data suggest that the regulation of expression of Cldns during osteoblast differentiation is complex and differentiation stage dependent.

### Regulation of Cldn-1 expression

**Figure 7:**
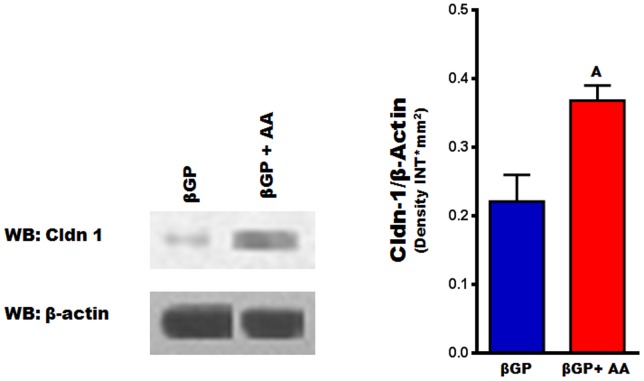
Cldn-1 protein levels during early stages of ascorbic acid induced osteoblast differentiation. Primary osteoblasts isolated from calvarias were treated with βGP ± AA for 6 days, before Cldn-1 expression was evaluated by western blotting. Values (means ± SEM; n = 4). A = <0.05 vs. untreated cells (βGP alone).

**Figure 8:**
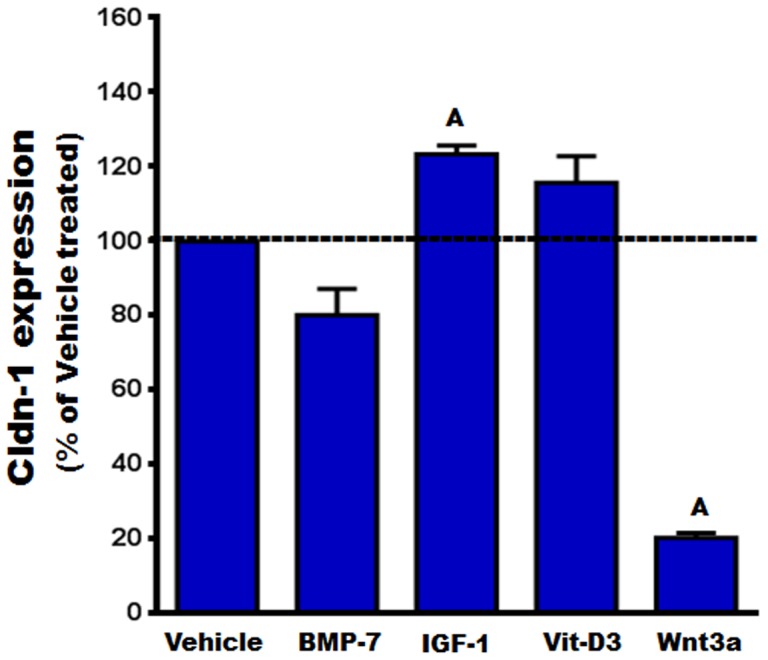
The regulation of Cldn-1 expression. MC3T3-E1 cells were treated with vehicle, BMP-7, IGF-1, vitamin-D3 (Vit-D3), and Wnt3a for 72 hrs, before the expression level of Cldn-1 was examined by real time RT-PCR. Values (means ± SEM; n = 4) are represented as % vehicle-treated control. A = <0.05 vs. vehicle control.

Among the several Cldns expressed in osteoblasts, Cldn-1 is of particular interest because it is known to be regulated by the key bone formation regulator IGF-1 [29], and its expression, as shown by our data, increased by more than 10 fold during osteoblast differentiation (Fig. 5B). We next sought to confirm that the increase in mRNA level of Cldn-1 during early stages (between day 6 and 8) of osteoblast differentiation was commensurate with an increase at the protein level using western blot analysis. Figure 7 shows that AA treatment for 6 days caused a 2-fold increase in Cldn-1 protein levels in newborn mouse calvarial osteoblasts. Osteoblasts are known to produce a number of growth factors which act in an autocrine/paracrine manner to regulate proliferative and differentiative functions [8]. In order to determine if bone growth factors regulate Cldn-1 expression, Cldn-1 mRNA level was measured after treating MC3T3-E1 mouse preosteoblast cells with different osteoregulatory agents (BMP-7, IGF-1, vitamin-D3, and Wnt3a) for 72 hours. As expected and previously reported, IGF-1 (30 ng/ml) treatment increased the expression of Cldn-1 in MC3T3-E1cells [29], whereas treatment with Wnt3a (10 ng/ml) decreased Cldn1 expression by 80% (Fig. 8). On the other hand, Cldn-1 expression was not affected by BMP-7 (30 ng/ml) or vitamin-D3 (10 nM) treatment (Fig. 8).

### The effect of knocking down Cldn-1 expression on MC3T3-E1 cell proliferation and differentiation

**Figure 9:**
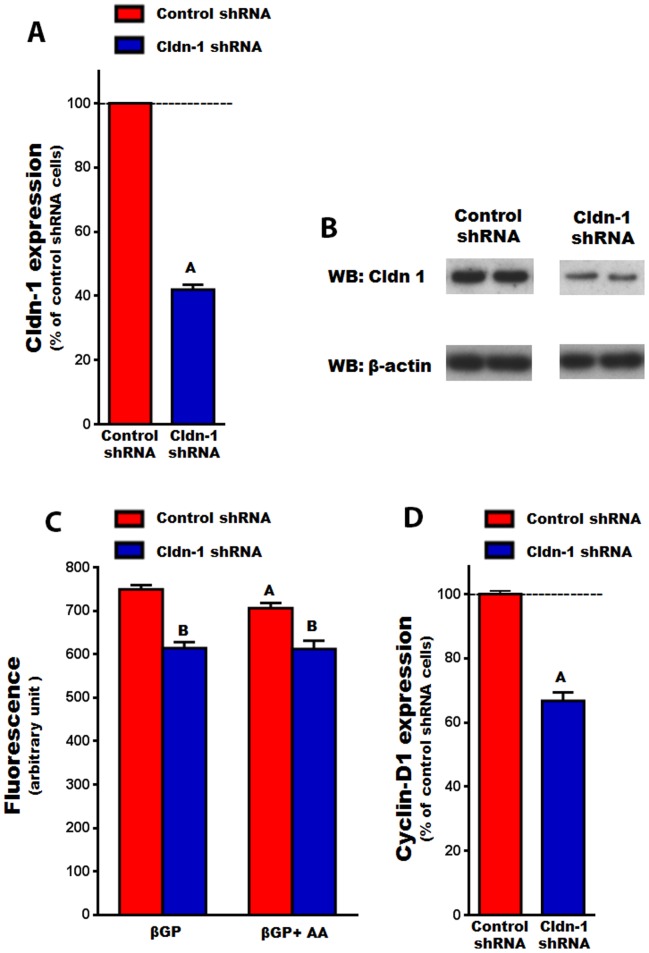
The effect of Cldn-1 knockdown on osteoblast proliferation in MC3T3-E1 cells. A) Cldn-1 expression in MC3T3-E1 cells transduced with control shRNA or Cldn-1 shRNA as determined by real time RT-PCR. Values (means ± SEM; n = 4). A = <0.0001 vs. control shRNA. B) Cldn-1 protein level in MC3T3-E1 cells transduced with control shRNA or Cldn-1 shRNA as determined by western blot using whole cell lysates. C) Cell proliferation: MC3T3-E1 cells transduced with control or Cldn-1 shRNA and treated with βGP ± AA for 48 hrs, before cell proliferation was assessed using the CYQUANT© cell proliferation kit by measuring fluorescence after excitation at 485 nm and by emission detection at 530 nm. Values (means ± SEM; n = 8). A = <0.05 vs. βGP treated, and B = <0.05 vs. control shRNA at corresponding treatment. D) The expression of a cell proliferation marker gene (cyclin-D1) in MC3T3-E1 cells transduced with control shRNA or Cldn-1 shRNA and treated with βGP ± AA for 24 hrs, as determined by real time RT-PCR. Values (means ± SEM; n = 4) are presented as % of control shRNA. A = <0.05 vs. control shRNA cells.

Cldn-1 is known to regulate cell proliferation and differentiation in a variety of cell types [30]–[32]. Therefore, the role of Cldn-1 in regulating osteoblast proliferation and differentiation was determined by knocking it down, using lentivirus shRNA, in MC3T3-E1 cells. First, we confirmed that MC3T3-E1 cells express Cldn-1 and that its expression was increased by 3 fold (P<0.01) by AA treatment at day 6 (data not shown). It was observed that Cldn-1expression was significantly decreased at both the mRNA and protein levels, in cells expressing Cldn-1 shRNA compared to control shRNA (Fig. 9A, B). It was found that cell proliferation was reduced by 18% and 13%, compared to control shRNA cells, when treated with βGP or AA, respectively (Fig. 9C). Moreover, in agreement with reduced cell proliferation in Cldn-1 shRNA cells, the expression of the osteoblast proliferation marker gene cylcin-D1 was reduced by 41% (Fig 9D).

**Figure 10:**
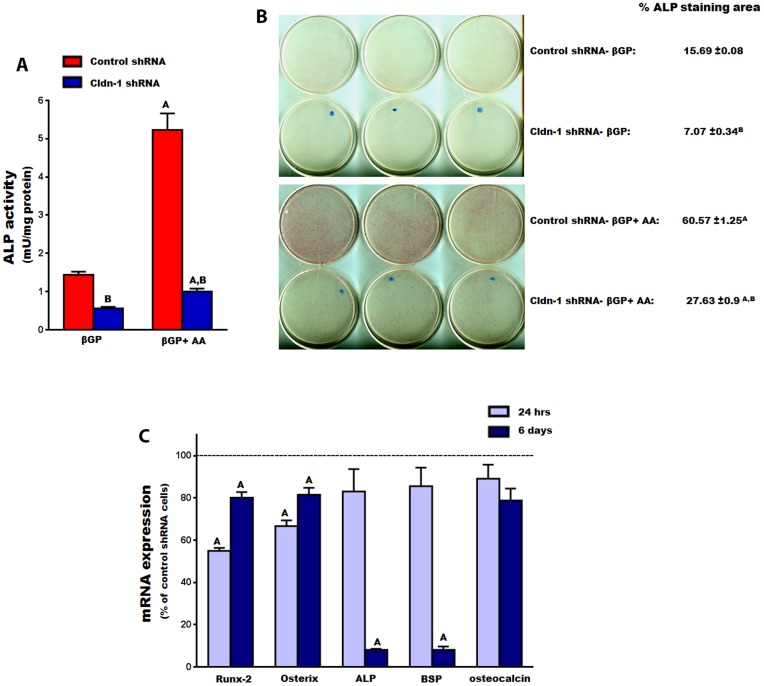
The effect of Cldn-1 knockdown on osteoblast differentiation in MC3T3-E1 cells. A) ALP activity was determined in MC3T3-E1 cells transduced with control or Cldn-1 shRNA and treated with βGP ± AA for 72 hrs. Values (means ± SEM; n = 8). A = <0.05 vs. βGP treated, and B = <0.05 vs. control shRNA at the corresponding treatment. B) ALP staining was determined on MC3T3-E1 cells transduced with control or Cldn-1 shRNA and treated with βGP ± AA for 6 days, followed by ALP activity staining. Values (means ± SEM; n = 5) are represented as % ALP stained area. A = <0.05 vs. βGP treated, and B = <0.05 vs. control shRNA at the corresponding treatment. C) The expression of osteogenic master transcription factor genes (osterix and Runx-2) and osteogenic marker genes (ALP, bone sialoprotein (BSP), and osteocalcin) was evaluated in control and Cldn-1 shRNA MC3T3-E1 treated β-glycerophosphate (βGP) with or without ascorbic acid (AA) for 24 hrs and 6 days using real time RT-PCR. Values (means ± SEM; n = 4) are presented as % of control shRNA. A = <0.05 vs. control shRNA cells.

Given that Cldn-1 expression is upregulated during early stages of osteoblast differentiation, we examined whether it plays a role in regulating expression of osteoblast differentiation markers. Knockdown of Cldn-1 expression in MC3T3-E1 cells reduced ALP activity by 61% (Fig. 10A). AA treatment caused a 4-fold increase in ALP activity in control shRNA treated cells, as expected. However, the effect of AA on ALP activity was reduced by 80% upon Cldn-1 knockdown (Fig. 10A). Consistent with these data, inhibition of Cldn-1 reduced ALP staining, in cells treated with AA (Fig. 10B).

To further characterize the positive effect of Cldn-1 on osteoblast differentiation, mRNA levels of osteoblast specific differentiation markers were determined in control and Cldn-1 shRNA cells, treated with or without AA. While the mRNA levels of ALP and bone sialoprotein were not found to be significantly decreased in Cldn-1 shRNA compared to control 24 hrs after AA treatment, their levels were decreased by 92% 6 days after AA treatment (Fig. 10C). Even though knocking down Cldn-1 appeared to reduce osteoclacin expression (a late osteoblast differentiation marker gene) at these time points, the difference did not reach statistical significance (Fig. 10C). Finally, the expression levels of transcription factors that are required for osteoblast differentiation was evaluated in control and Cldn-1 shRNA cells. We found that down regulating Cldn-1 expression reduced the expression of both osterix and Runx-2 mRNA levels, by 33% and 45% respectively, when treated with AA for 24 hrs (Fig. 10C). This effect was still maintained in the Cldn-1 shRNA cells, even after 6 days of AA treatment, albeit to a lower extent (Fig. 10C).

### The effect of knocking down Cldn-1 expression on β-catenin levels

**Figure 11:**
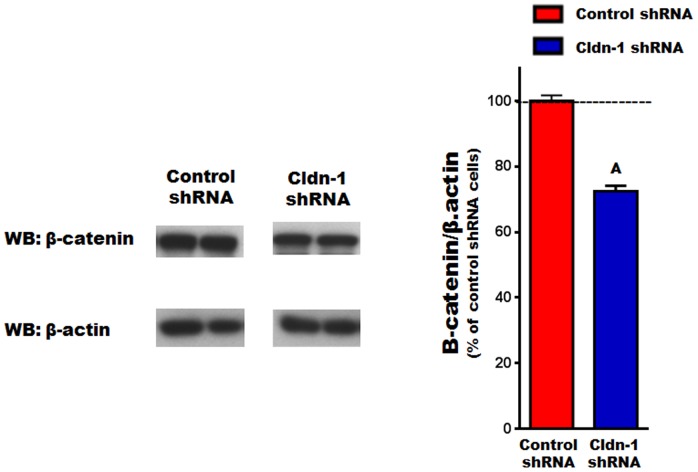
The effect of Cldn-1 knockdown on β-catenin. Control shRNA or Cldn-1 shRNA MC3T3-E1 cells were treated with AA for 48 hrs, then β-catenin expression was evaluated by western blotting using whole cell lysates. Values (means ± SEM; n = 4). A = <0.05 vs. control shRNA cells.

To gain more insight into the molecular pathway(s) underlying Cldn-1 function in osteoblasts, we tested the effect of its knockdown on β-catenin signaling. This is based on β-catenin's established importance in regulating osteoblastogenesis [33], [34], as well as reports that inhibition and overexpression of Cldn-1 in a colon cancer cell line leads to a reduction and activation of β-catenin signaling, respectively [35], [36]. Thus, β-catenin protein level was evaluated in Cldn-1 shRNA compared to control shRNA cells, treated with AA for 48 hrs. Indeed, we found that β-catenin protein level decreased by 28% as a consequence of Cldn-1 knockdown (Fig 11), suggesting that it may be involved in Cldn-1 regulation of osteoblastogenesis.

## Discussion

This study provides the first comprehensive investigation of the expression of Cldn family members during bone cell differentiation, in mice. Specifically, we report for the first time that primary mouse osteoclasts differentially express several Cldns in a time dependent manner. Furthermore, Cldns were found to be differentially expressed and highly regulated during primary osteoblast differentiation In an attempt to decode the function of Cldns in regulating skeletal development and maintenance, we focused on the role of Cldn-1 during osteoblastogenesis. We determined that Cldn-1 expression is regulated by several osteoregulatory agents such as IGF-1 and Wnt3a and that Cldn-1 is a positive regulator of osteoblast proliferation and differentiation. In addition, knockdown of Cldn-1 in osteoblasts leads to a reduction in β-catenin protein levels, suggesting that this pathway is possibly involved in Cldn-1-mediated modulation of osteoblastogenesis.

In agreement with the complex expression patterns of Cldns reported in different tissues, we found that Cldns also exhibit cell type and differentiation stage specific patterns of expression, during bone cells differentiation. As for osteoclasts, we were the first to provide evidence that Cldns, specifically Cldn-18, are expressed in osteoclasts [22]. Aside from Cldn-18, the expression profile of other Cldn family members has not been previously reported in the literature. In this regard, the current study demonstrates, for the first time, that several Cldns were expressed in primary osteoclasts, in a differentiation stage dependent fashion. Furthermore, three distinct expression profiles were observed during osteoclast differentiation: 1. upregulation or downregulation during early stages, suggesting that these Cldns may have potential functions during the proliferation stage; 2. up regulation during late stages, supporting possible roles in regulating osteoclast function and activity; and 3. no apparent change in the expression of some Cldns, which may indicate a lack of a role during osteoclastogenesis. Regarding their function, Cldn-18 is the only family member shown to play a role in osteoclastogenesis [22]. While these studies clearly document a critical non-canonical role for Cldn-18 in regulating osteoclast differentiation, the current study suggests that other Cldn family members may also have potential roles in regulating these processes. In addition, the issue of whether Cldn-18 interacts with other Cldns to regulate bone resorption remains to be elucidated.

As for osteoblasts, their differentiation processes *in vitro* are divided into several stages and regulated by differential and sequential expression of several genes [7], [8]. While rat osteoblasts are known to express several Cldns [20], [21], the consequence of cellular differentiation on Cldn expression has not been documented. Our study revealed for the first time that the expression pattern of Cldns may be osteoblast differentiation stage-dependent. Many Cldns showed an increase in their expression levels, some were found to be downregulated, and others exhibited no change, indicating possible diverse roles for Cldns during different stages of osteoblastogenesis. Consistent with our finding, it has been shown that the expression of Cldn-1 and -2 was higher in osteoblast like MC3T3-E1 cells compared to osteocyte-like MLO-Y4 cells [29]. By contrast, an earlier study showed that Cldn-1 and Cldn-2 mRNA levels were upregulated during the mineralization stage compared to the proliferation stage [20]. While expression levels of several Cldns seem to vary depending on differentiation stage of osteoblasts, we have not determined whether these changes are biologically significant. Therefore, our future experiments will evaluate the biological role of the various Cldn family members in regulating osteoblast proliferation/differentiation to determine if their differentiation stage-dependent expression has physiological relevance. In-line with differentiation stage dependent patterns of Cldns expression, it has been shown that other cells of mesenchymal origin differentially express Cldns [37], [38]. For example, Cldn-6 was found to be upregulated and plays an important role during adipocyte differentiation [37]. Moreover, our findings that all “classic” Cldns mRNA levels were increased during osteoblast differentiation suggest that these closely related Cldns may exert similar/redundant functions, and possibly regulated in a similar manner. By contrast, the diverse expression patterns observed with the “non-classic” Cldns supports the idea that they may have district functions, and may, therefore, be regulated differently. Thus, even though these data suggest potential roles of Cldn family members in regulating osteoblastogenesis, our future studies will examine their role in skeletal development and maintenance, by employing both gain and loss of function experiments *in vivo* and *in vitro*.

In terms of the regulation of Cldn expression, multiple studies in other tissues have shown that Cldn expression and function is regulated by a host of growth factors, hormones, and cytokines [14]. The observed complex expression patterns of Cldns during osteoblast differentiation appear to indicate that Cldn expression is tightly regulated. Thus, these tight regulation processes of Cldn expression during AA induced osteoblast differentiation could be explained by either direct effects of AA treatment and/or indirect effects. The latter could be mediated by growth factors and/or bone matrix proteins secreted during osteoblast differentiation, which, in turn, act in an auto/paracrine fashion. In order to better understand the regulation and function of Cldns in bone, our efforts focused on Cldn-1 based on its interesting differential expression pattern, (i.e., upregulation of Cldn-1 during early stages of osteoblast differentiation, whereas downregulation during late stages); and its established importance in tissue development as mice with targeted disruption of the Cldn-1 gene failed to survive after birth [39]. Consistent with an important role for Cldn-1 are our findings that its expression is regulated by IGF-1 and Wnt3a. The increased expression of Cldn-1 following IGF-1 treatment is similar to a previous report in which IGF-1 was found to upregulate Cldn-1 expression, via a MAPK dependent pathway (25). On other hand, Wnt3a, a known stimulator of bone formation, significantly decreased the expression of Cldn-1. Interestingly however, an opposite regulation pattern was observed in colon cancer cells supporting the notion that Cldns regulation by Wnt signaling may be tissue/cell type specific [40], [41].

Regarding Cldn-1 function, it has been shown that Cldn-1 acts canonically as a cation restrictive barrier in epithelial as well as endothelial tissues [10], [42]. Aside from this function, there is substantial evidence that Cldn-1 participates in intracellular signaling that controls cell proliferation and differentiation [30]–[32]. While the canonical function of Cldn-1 in bone cells has been suggested in the literature, virtually nothing is known about its non-canonical function, i.e., as a mediator of cell signaling, in such cells [29]. Consequently, knocking down Cldn-1 reduced osteoblast proliferation and the expression of the cell proliferation marker gene cyclin-D1, indicating that Cldn-1 promotes osteoblast proliferation. In addition, several studies in other cell types have demonstrated that Cldn-1 is a promoter, inhibitor, or has no effect on cell proliferation, thereby supporting the notion that the “proliferative” function of Cldn-1 is cell type-dependent [31], [32], [35]. Besides regulating proliferation, Cldn-1 appears to play an important role in regulating early stage osteoblast differentiation as revealed by data from Cldn-1 knockdown experiments. In agreement with the observed upregulation of Cldn-1 during early stages of osteoblast differentiation, Cldn-1 deficiency had no significant effect on the expression of the late stage osteogenic marker gene osteocalcin. In addition, previous studies demonstrated that Cldn-1 can modulate the expression of transcription factors in various tissues [36], [43], [44]. Accordingly, we determined if Cldn-1 regulates the expression of Runx-2 and osterix, master transcription factors of osteoblast differentiation, and found their levels to be reduced significantly as a consequence of Cldn-1 knockdown. It seems that Cldn-1 regulates the expression of transcription factors during early time points, which seems to be consistent with their consequent regulation of the expression of osteogenic marker genes. The finding that the effect of Cldn-1 knockdown on expression of osteogenic transcription factors was maintained after 6 days, suggests that Cldn-1 deficiency inhibits osteoblast differentiation. However, the issue whether inhibition of Cldn-1prevents or delays differentiation will be addressed in future studies. Together these findings underscore Cldn-1 as a positive regulator of (early) osteoblastogenesis, which is consistent with its reported function in early dentinogenesis [30]. On the contrary, it has been reported that Cldn-1 inhibits differentiation of other cell types [32], thereby, providing evidence that its regulation of differentiation is cell type and stage dependent.

In terms of potential mechanism(s) for Cldn-1 actions, we evaluated the effect of Cldn-1 knockdown on β-catenin, which is a major pathway known to be critical for osteoblastogenesis [33], [34]. We found that knockdown Cldn-1 in osteoblasts leads to a reduction in β-catenin protein levels. In support of our findings, it has been reported that inhibition and overexpression of Cldn-1 in a colon cancer cell line leads to reduction and activation of β-catenin signaling, respectively [35], [36]. However, the detailed mechanism by which Cldn-1 modulates β-catenin signaling remains to be determined, and the cause and effect relationship between reduced β-catenin level and reduction in proliferation/differentiation caused by Cldn1 knockdown in osteoblasts will be the focus of future experiments. Nonetheless, one of the potential mechanisms may be through phosphorylation/inactivation of GSK3, which normally results in β-catenin degradation. Another possible mechanism is by direct binding, which will make β-catenin less accessible to the destruction complex [35]. On the other hand, Cldn-1 itself has been found to be a target for β-catenin signaling in colon cancer [40]. Therefore, we cannot exclude the possibility that β-catenin signaling is also upstream of Cldn-1 and/or there exists some kind of cross talk or feedback loop between them in regulating osteoblastogenesis. While our data suggests that Cldn-1 may regulate β-catenin signaling, we cannot exclude the involvement of other pathways such as TGF-β, BMP, and Notch signaling. Thus, the delineation of the molecular pathway(s) by which Cldn-1 acts should advance our understanding of the regulation of osteoblast differentiation.

In conclusion, here we show for the first time that Cldn family members are expressed and tightly regulated in both primary osteoblasts and osteoclasts, in a differentiation stage dependent manner. Furthermore, we provide compelling evidence that Cldn-1 is a novel positive regulator of osteoblast differentiation and proliferation, and its regulation is complex and mediated by several osteoregulatory factors. Collectively, the observed complexity in the expression patterns of Cldns during bone cells differentiation, and the finding that Cldn-1 regulates osteoblast differentiation suggests that Cldns may have potential roles in regulating bone homeostasis. Finally, our future understanding of how Cldns regulate osteoblast and osteoclast function, and overall bone homeostasis could lead to the development of Cldn-based drug targets for diagnosis and therapeutic management of osteoporosis.
